# Identification of High-Yielding Iron-Biofortified Open-Pollinated Varieties of Pearl Millet in West Africa

**DOI:** 10.3389/fpls.2021.688937

**Published:** 2021-09-22

**Authors:** Prakash I. Gangashetty, Mohammed Riyazaddin, Moussa Daouda Sanogo, Drabo Inousa, Kassari Ango Issoufou, Peter A. Asungre, Ousmane Sy, Mahalingam Govindaraj, Angarawai Ijantiku Ignatius

**Affiliations:** ^1^International Crops Research Institute for the Semi-Arid Tropics, (ICRISAT), Patancheru, India; ^2^International Crops Research Institute for the Semi-Arid Tropics, (ICRISAT), Niamey, Niger; ^3^Institut d’Economie Rurale, Cinzana, Mali; ^4^Institut de l’Environnement et du Recherches Agricoles de Burkina Faso (INERA), Ouagadougou, Burkina Faso; ^5^Institut National de la Recherche Agronomique du Niger (INRAN), Maradi, Niger; ^6^CSIR-Savanna Agriculture Research Institute, Bawku, Ghana; ^7^Senegalese Institute of Agricultural Research (ISRA), Bambey, Senegal; ^8^International Crops Research Institute for the Semi-Arid Tropics, (ICRISAT), Kano, Nigeria

**Keywords:** pearl millet, micronutrient malnutrition (MNM), CHAKTI, AMMI analysis, grain Fe and Zn

## Abstract

Pearl millet is a predominant food and fodder crop in West Africa. This study was carried out to test the newly developed open-pollinated varieties (OPVs) for field performance and stability for grain yield, grain iron (Fe), and grain zinc (Zn) contents across 10 locations in West Africa (i.e., Niger, Nigeria, Mali, Burkina Faso, Senegal, and Ghana). The test material consisted of 30 OPVs, of which 8 are Fe/Zn biofortified. The experiment was conducted in a randomized complete block design in three replications. ANOVA revealed highly significant variability for grain yield and micronutrient traits. The presence of genotype × environment (G × E) indicated that the expressions of traits are significantly influenced by both genetic and G × E factors, for grain Fe and Zn contents. Days to 50% flowering and plant height showed less G × E, suggesting these traits are largely under genetic control. The genotypes CHAKTI (46 days), ICTP 8203 (46 days), ICMV 177002 (50 days), ICMV 177003 (48 days), and Moro (53 days) had exhibited early flowering across locations leading to early physiological maturity. CHAKTI (1.42 t/ha yield; 62.24 mg/kg of grain Fe, 47.29 mg/kg of grain Zn) and ICMP 177002 (1.19 t/ha yield, 62.62 mg/kg of grain Fe, 46.62 mg/kg of grain Zn) have performed well for grain yield and also for micronutrients, across locations, compared with the check. Additive Main Effect and Multiplicative Interaction (AMMI) ANOVA revealed the highly significant genotypic differences, the mean sum of squares of environment, and its interaction with the genotypes. Based on the AMMI stability value (ASV), the most stable genotype is SOSAT-C88 (ASV = 0.04) for grain yield and resistance to downy mildew; mean grain yield and stability rankings (YSI) revealed that the genotypes CHAKTI, SOSAT-C88, and ICMV IS 99001 were high yielding and expressed stability across regions. The strong correlation (*r* = 0.98^∗∗^) of grain Fe and Zn contents that merits Fe-based selection is highly rewarding. CHAKTI outperformed over other genotypes for grain yield (71% higher), especially with early maturing varieties in West Africa, such as GB 8735, LCIC 9702, and Jirani, and for grain Fe (16.11% higher) and Zn (7% higher) contents across locations, and made a candidate of high-iron variety to be promoted for combating the micronutrient malnutrition in West and Central Africa (WCA).

## Introduction

Pearl millet (*Pennisetum glaucum* L.) is an important food and fodder crop for people living in the semi-arid regions of Asia and Africa. The people living in the semi-arid regions mostly consume cereal-based foods and do not have access to diverse food, hence, leading to serious malnutrition in children and anemia in women (Underwood, 2000). Micronutrient malnutrition is a major concern in the developing world, where more than 2 billion people are malnourished (World Health Organization, 2006; Tulchinsky, 2010; Kramer and Allen, 2015; Webb et al., 2018). Malnutrition in Africa is a rising concern where children are affected by stunted growth and low IQ. Young children and pregnant women are most vulnerable to micronutrient deficiencies due to their rapid growth and development (Black et al., 2013; Saltzman et al., 2017). At least half of the children worldwide younger than 5 years of age suffer from vitamin and mineral deficiencies, while almost 2 in 3 children between 6 months and 2 years of age are not fed on food that supports their healthy development (UNICEF, 2019). Although only required in small amounts, micronutrients are not produced in the body and must be derived from the diet. It is severe in regions where people depend on staple crops, which are low in micronutrients, to meet their energy requirements. Hence, improving the micronutrient concentration of locally adapted staple food crops will be a sustainable solution to address this malnutrition. Efforts are being made to provide fortified foods to these vulnerable groups, which is a cost-effective strategy, but it is difficult to ensure the availability of these fortified foods to people living in remote areas. Food diversification is another tool to combat micronutrient malnutrition, but the high cost involved and limited food availability make it unsustainable.

Biofortification, the process of increasing the concentration of micronutrients in the staple crops to have a measurable impact on human nutrition after consumption, is therefore important for combating micronutrient malnutrition. Biofortification is becoming more of the breeding process by which the nutrient density is increased through conventional plant breeding and modern biotechnology without altering the preferred traits of the farmers. (1) Iron biofortification of beans, cowpea, and pearl millet, (2) zinc biofortification of maize, rice, and wheat, and provitamin A, and (3) carotenoid biofortification of cassava, maize, rice, and sweet potato are currently underway and at different stages of development (Saltzman et al., 2013). Biofortification has been regarded as a novel and sustainable vehicle in the reduction of micronutrient malnutrition (Bouis et al., 2011).

The HarvestPlus program of the CGIAR is supporting global biofortification efforts, through which the International Crops Research Institute for the Semi-Arid Tropics (ICRISAT) released the ever-first high grain Fe pearl millet variety “Dhanashakti” in 2014 by exploiting the intrapopulation variability (recurrent selection) within ICTP 8203 variety, an early open-pollinated variety (OPV) popularly grown in India (Rai et al., 1990; Govindaraj et al., 2019). Since then, pearl millet hybrids rich in kernel iron and zinc concentrations have been bred and released in India through conventional (i.e., heterosis) breeding methods (Govindaraj et al., 2018, 2019).

Many studies have shown the positive impact of the consumption of biofortified crops, and the results of these studies were also similar to iron fortification and supplementation strategies to fight against hidden hunger (Kodkany et al., 2013; Saltzman et al., 2013; Haas, 2014; Finkelstein et al., 2015). The use of biofortified pearl millet varieties could have a positive effect on Fe and Zn intakes in the long term. The studies conducted by Kodkany et al. (2013) revealed that the quantities of both iron and zinc were absorbed when iron- and zinc-biofortified pearl millet was fed to children aged 2 years as the major staple food and was more than adequate to meet the physiological requirements for these micronutrients.

The regions in West and Central Africa (WCA), where pearl millet is significantly grown and consumed, have no biofortified variety at present. The ICRISAT center in Niamey, Niger with the help of the HarvestPlus program employs in developing high-yielding biofortified pearl millet varieties and hybrids. WCA regions significantly hold the undernutrition populations, and it is very appropriate to breed, test, and identify the Fe/Zn-rich pearl millet varieties besides better yield and resistance to biotic stress across the region of WCA. Most of the pearl millet varieties existing with the farming community have lower levels of grain Fe (<30.0 mg/kg) and Zn (<30.0 mg/kg) contents. Hence, this study was conducted to identify the pearl millet varieties with high grain Fe and Zn contents, their stable performance across the region, and the influence of environmental factors on the inheritance of these traits.

## Materials and Methods

### Experimental Material and Design

The experimental material consisted of 30 pearl millet varieties, of which 8 varieties had high grain Fe and Zn contents, 20 with moderate levels of grain Fe and Zn, and 2 local checks GB 8735 and Jirani. GB 8735 is the highest acreage cultivated in Niger, Togo, and adjoining countries with its early maturity and stability. This variety was also noticed with higher Fe/Zn in the regions. Using this variety for both early maturity and micronutrient is highly appropriate in this study. The trials were conducted in 10 locations of WCA representing the Sahelian agroecology with an annual rainfall of 400 mm. The test locations were Niger (Sadore, Konni, Maradi, and Magaria), Nigeria (Kano), Mali (Cinzana), Burkina Faso (Gampella), Ghana (Bawku), and Senegal (Bambey and Nioro) in WCA. The material was sown in randomized complete block design in three replications in the rainy season of 2018. A basal dose of fertilizer was applied to the field at the rate of 100 kg/ha. The genotypes were sown in two-row plots with a row length of 3.0 m long and a row-to-row distance of 0.80 and 0.20 m in between the plants. Thinning of the test entries to one plant per hill was carried out at 15–21 days after seedling emergence (DAE). Hand weeding of the test plots was carried out when necessary. Microdosing with urea was carried out at 30 DAE.

Data on agronomic and morphological traits were recorded in the test plots. Data on days to 50% flowering, plant height (cm), number of downy mildew plants, panicle length (cm), circumference (cm), and grain yield (t/ha) were recorded. Downy mildew incidence were calculated based on the percentage incidence using the number of plants affected to the total number of plants in the plot multiplied by 100. Grain samples for Fe and Zn analysis were collected before harvesting on the standing crop eliminating the plants grounded and contaminated with soil. Proper care was taken in cleaning the grain samples for analysis to avoid contamination from metals and dust.

### Micronutrient Estimation

Pearl millet grain micronutrient estimation was carried out using the energy dispersive X-ray fluorescence (EDXRF) (Paltridge et al., 2012a,b; Guild and Stangoulis, 2016) method. The EDXRF analysis was carried out using Oxford X-Supreme 8000 (Oxford Instruments plc, United Kingdom) with a 10-sample carousel. Analyses were conducted in supplied sample cups prepared as reported previously (Paltridge et al., 2012a,b; Guild and Stangoulis, 2016), with a 4-μm Poly-4 XRF sample film used to seal one end of the cup. Sample cups were cleaned and prepared before each analysis to minimize cross-contamination between samples. Samples of >5 g were used for all analyses to ensure that samples were “infinitely thick” in terms of EDXRF analysis (Paltridge et al., 2012b). Calibrations for Fe and Zn in pearl millet were achieved using 20 standards based on the data of inductively coupled plasma optical emission spectrophotometry (ICP-OES).

### Statistical Analysis

The replicated data from each location are subjected to perform ANOVA and Additive Main Effects and Multiplicative Interaction (AMMI) analysis using GenStat software version 18.0 (GenStat, 2015). The significant differences between the genotypes were tested by using the *F*-test, and the genotypic means were compared by the least significant difference at *p* ≤ 0.05. AMMI analysis permits the estimation of the interaction effect of a genotype in each environment, and it helps to identify genotypes best suited for specific environmental conditions. The trait phenotypic correlation coefficient was analyzed using Excel. Heritability estimates were calculated using *H*^2^ = *V*_*g*_/*V*_*p*_, where *H*^2^ is the heritability estimate; *V*_*g*_, the variation in genotype; and *V*_*p*_, the variation in phenotype.

## Results

The ANOVA has revealed a highly significant (*P* ≤ 0.01) mean sum of squares of entries, locations (hereafter referred to as environment), and genotypes × location [hereafter referred to as genotype × environment (G × E)] for all the studied traits (Table 1). The mean sum of squares of genotypes was higher than the mean sum of squares of environments. The G × E influenced the trait determination in these 10-environment testing; however, it was very much lower in variance magnitude for all the traits. For instance, flowering, grain yield, and grain Fe and Zn contents were largely controlled by genotypic variance. Days to 50% flowering showed 29 times higher mean square magnitude than the G × E source of variation. The heritability for agronomic and morphological traits revealed presence of high heritability for all the studied traits. The estimation of genetic advance percentage mean showed high genetic advance for downy mildew resistance (106.15%), panicle length (66.50%), grain yield (49.32%), and grain Fe content (39.01%).

**TABLE 1:** Mean sum of squares of pearl millet genotypes across locations in West Africa.

**Sources of variation**	**df**	**Downy mildew damage**	**Days to 50% flowering**	**Plant height**	**Panicle length**	**Panicle circumference**	**Grain yield**	**Grain Fe content**	**Grain Zn content**
Replication	2	363.00	11.81	616.50	61.34	4.58	0.02	12.72	36.54
Environment	9	9924.10**	660.53**	7793.60**	334.48**	14.65**	1.62**	197.27**	361.60**
Genotype	29	1369.10**	1628.99**	26451.30**	3316.05**	47.92**	1.75**	2125.37**	689.32**
Environment × genotype	260	296.50**	56.04**	757.00**	80.04**	1.40**	0.12**	27.43**	28.12**
Residual	574	102.90	9.61	233.90	15.36	1.04	0.02	8.95	12.39
Total	892								
Heritability (H^2^)		80.21	96.62	97.18	97.62	97.09	93.39	98.72	95.98
Genetic advance as % mean		106.15	26.92	29.62	66.50	28.16	49.32	39.01	25.81

**Mean sum of squares significant at *P* = 0.01.

The mean performance of the pearl millet genotypes indicated significant *F* probabilities (*P* ≤ 0.01) for all the studied traits (Table 2). The genotypes CHAKTI, Faringuero, IBMV 8402, ICMV 167005, ICMV 167006, ICMV 177001, ICMV 177002, ICMV 177003, ICMV 177004, ICMV IS 85327, ICMV IS 99001, ICTP 8203, Moro, SARIA-1, SOSAT-C88, Souna 3, and WAAP-NAARA had shown lower (<10%) downy mildew damage percentage when compared with the susceptible check, Sadore local (17.69 %).

**TABLE 2:** Mean performance of the pearl millet (*Pennisetum glaucum* L.) genotypes across locations in West Africa.

**Genotype**	**Downy mildew damage%**	**Days to 50% flowering**	**Plant height (cm)**	**Panicle length (cm)**	**Panicle circumference (cm)**	**Grain yield (t/ha)**	**Grain Fe content (mg/kg)**	**Grain Zn content (mg/kg)**
AFRIBEH-NAARA	12.09	47.43	190.00	24.03	9.30	0.79	45.04	38.60
AKAD-KOM	15.92	48.03	177.40	17.56	10.45	0.83	52.66	42.39
CHAKTI	1.97	45.57	168.50	23.78	9.59	1.42	62.24	47.29
Faringuero	9.57	58.87	208.20	35.58	8.99	0.73	34.55	32.31
Gamoji	11.13	62.57	231.40	41.10	9.91	1.19	37.29	32.82
GB 8735	17.22	49.43	170.30	23.39	10.22	0.83	53.60	44.02
IBMV 8402	8.29	61.97	210.30	39.02	7.83	0.99	38.21	33.86
ICMP 177001	23.53	48.63	171.30	23.71	10.53	0.85	57.67	43.77
ICMP 177002	26.25	48.37	173.20	25.24	10.03	1.19	62.62	46.62
ICMV 167005	3.27	65.63	240.60	44.28	8.08	1.31	38.12	35.70
ICMV 167006	6.88	59.43	231.50	32.10	6.95	1.13	42.41	35.51
ICMV 177001	4.14	65.73	256.70	29.61	7.19	0.61	37.51	32.41
ICMV 177002	3.74	50.07	195.30	26.84	9.34	0.83	43.72	36.89
ICMV 177003	9.92	48.40	168.40	23.83	10.29	0.83	52.17	40.79
ICMV 177004	9.82	56.23	179.00	32.97	9.12	0.80	40.70	36.01
ICMV 221	11.56	44.57	163.70	22.93	10.12	0.79	51.36	42.63
ICMV 167001	23.01	49.07	176.60	29.46	10.96	1.33	50.52	40.23
ICMV IS 85327	10.94	59.87	234.10	51.06	7.93	0.97	37.05	33.00
ICMV IS 94222	11.88	60.57	241.70	60.01	7.80	1.28	35.60	32.86
ICMV IS 99001	5.63	58.93	218.00	51.12	7.87	1.32	33.34	30.24
ICTP 8203	9.95	45.47	168.80	24.79	10.29	0.75	55.07	43.19
Jirani	12.66	45.73	172.20	22.64	8.35	0.84	47.18	39.93
KAANATI	20.12	54.43	190.30	28.61	8.60	0.69	43.04	35.81
LCIC 9702	12.02	49.10	184.40	30.09	8.66	0.87	43.59	35.57
Moro	8.93	53.00	209.80	28.38	7.17	1.06	45.33	37.43
Sadore Local	17.69	66.60	235.50	46.46	7.29	0.73	33.71	29.84
SARIA-1	4.24	69.90	260.80	30.58	7.81	0.61	43.61	37.30
SOSAT-C88	1.19	58.27	221.60	28.19	11.11	1.38	36.64	33.16
Souna 3	2.26	60.30	221.40	47.95	7.59	0.98	38.39	34.91
WAAPP-NAARA	2.34	56.00	192.90	26.03	10.09	0.88	37.50	33.56

Mean	11.00**	55.00**	202.00**	32.00**	9.00**	0.96**	44.00**	37.00**
Vr	13.31	169.49	113.07	215.90	45.99	88.67	237.47	55.65
SE ±	1.90	0.60	2.80	0.70	0.20	0.03	0.50	0.60
LSD	5.10	1.60	7.80	2.00	0.50	0.07	1.50	1.80
CV (%)	95.60	5.60	7.60	12.10	11.40	14.60	6.70	9.40

**F probabilities significant at P = 0.01; Vr, variance ratio; SE, standard error; LSD, least significant difference; CV, coefficient of variation.

The genotypes CHAKTI, ICMV 221, ICTP 8203, and Jirani had attained days to 50% flowering within 45 days, which is very early (Table 2). Other 11 genotypes also exhibited lower days to 50% flowering when compared with the overall mean (55.00 days) and with the local check (66.60 days). The mean plant height of 202.00 cm is recorded across locations. Fourteen genotypes exhibited greater plant height when compared with the mean plant height. Most of the genotypes with the lower days to 50% flowering exhibited moderate plant height.

The test genotypes exhibited a mean panicle length of 32.00 cm across locations (Table 2). The genotypes Gamoji (41.10 cm, 9.91 cm; panicle length and panicle circumference, respectively) and ICMV 177004 (32.97, 9.12) have shown greater panicle length and circumference across locations. The genotype ICMV IS 94222 had exhibited the highest panicle length of 60.01 cm, and AKAD-KOM has the least panicle length of 17.56 cm.

The mean performance of genotypes for grain yield across locations is given in Table 2. The overall mean for grain yield is 0.96 t/ha. The genotype CHAKTI (1.42 t/ha) has performed well for grain yield when compared with the other genotypes. The genotypes Gamoji, IBMV 8402, ICMP 177002, ICMV 167005, ICMV 167006, ICMV 167001, ICMV IS 85327, ICMV IS 94222, ICMV IS 99001, SOSAT-C88, and Souna 3 yielded high across locations when compared with the check Sadore local (0.73 t/ha).

The genotypes AFRIBEH-NAARA, AKAD-KOM, GB 8735, ICMP 177001, ICMV 177003, ICMV 221, ICTP 8203, and Jirani exhibited high grain Fe and Zn contents across locations when compared with the check Sadore local (33.71 mg/kg Fe and 29.84 mg/kg Zn), expressing lower than the overall mean (Table 2). The genotypes CHAKTI and ICMP 177002 have the highest grain Fe content of >62.0 mg/kg and grain Zn content of >45.0 mg/kg.

The AMMI ANOVA carried out to partition the total variances into its components revealed existence of highly significant (*P* ≤ 0.01) genotypic differences among all the traits (Table 3). The mean sum of squares of the environment and its interaction with the genotypes were also significant at *P* ≤ 0.01. The G × E interaction was further partitioned into IPCA 1 and IPCA 2, which were significant at *P* ≤ 0.01 for all the studied traits. The mean sum of squares of G × E interactions was lower than the mean sum of squares of the genotypes.

**TABLE 3:** Analysis of variance for AMMI model of pearl millet genotypes across locations in West Africa.

**Source**	**df**	**Downy mildew damage**	**Days to 50% flowering**	**Plant height**	**Panicle length**	**Panicle circumference**	**Grain yield**	**Grain Fe content**	**Grain Zn content**
Treatments	299	689.00**	226.80**	3461.00**	401.60**	6.31**	0.33**	236.00**	102.30**
Genotypes (G)	29	1369.00**	1629.00**	26451.00**	3316.10**	47.92**	1.75**	2125.40**	689.30**
Environments (E)	9	9924.00**	660.50**	7794.00**	334.50**	14.65**	1.62**	197.30**	361.60**
Block	20	305.00**	10.80^NS^	394.00*	19.90^NS^	1.88*	0.02^NS^	41.00**	28.10**
G × E	260	296.00**	56.00**	757.00**	80.00**	1.40**	0.12**	27.40**	28.10**
IPCA 1	37	1067.00**	248.50**	2345.00**	270.60**	3.18**	0.35**	53.30**	61.50**
IPCA 2	35	496.00**	49.90**	1099.00**	164.80**	3.14**	0.12**	45.80**	40.70**
Residuals	188	108.00	19.50	383.00**	27.00	0.73	0.08	19.00	19.30
Error	574	103.00	9.60	234.00	15.40	1.04	0.02	9.00	12.40

*,**F probabilities significant at P = 0.05 and 0.01, respectively; NS, non-significant probabilities; IPCA 1, interaction principal component axis one; IPCA 2, interaction principal component axis two.

**FIGURE 1:**
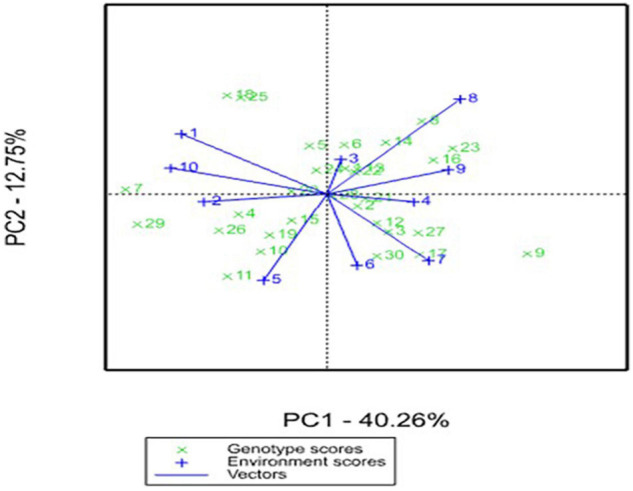
Additive Main Effects and Multiplicative Interaction (AMMI) biplot for grain yield across environments in West and Central Africa. 1. AFRIBEH-NAARA, 2. AKAD-KOM, 3. CHAKTI, 4. Faringuero, 5. Gamoji, 6. GB 8735, 7. IBMV 8402, 8. ICMP 177001, 9. ICMP 177002, 10. ICMV 167005, 11. ICMV 167006, 12. ICMV 177001, 13. ICMV 177002, 14. ICMV 177003, 15. ICMV 177004, 16. ICMV 221, 17. ICMV 167001, 18. ICMV IS 85327, 19. ICMV IS 94222, 20. ICMV IS 99001, 21. ICTP 8203, 22. Jirani, 23. KAANATI, 24. LCIC 9702, 25. Moro, 26. Sadore Local, 27. SARIA-1, 28. SOSAT-C88, 29. Souna 3, 30. WAAPP-NAARA.

**FIGURE 2:**
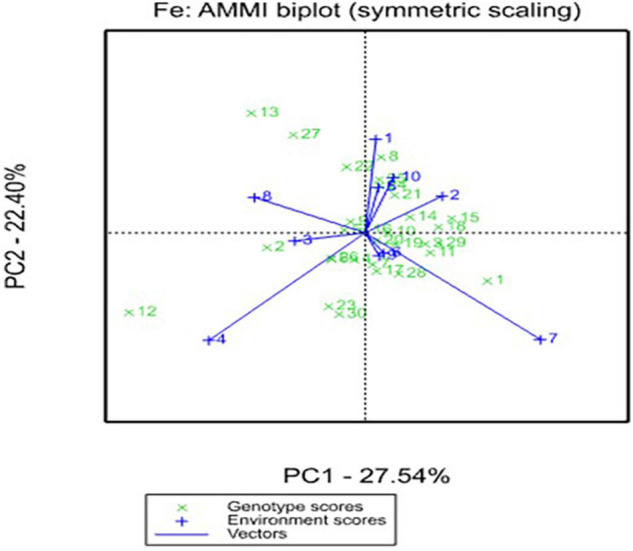
Additive Main Effects and Multiplicative Interaction (AMMI) biplot for grain Fe content across environments in West and Central Africa. 1. AFRIBEH-NAARA, 2. AKAD-KOM, 3. CHAKTI, 4. Faringuero, 5. Gamoji, 6. GB 8735, 7. IBMV 8402, 8. ICMP 177001, 9. ICMP 177002, 10. ICMV 167005, 11. ICMV 167006, 12. ICMV 177001, 13. ICMV 177002, 14. ICMV 177003, 15. ICMV 177004, 16. ICMV 221, 17. ICMV 167001, 18. ICMV IS 85327, 19. ICMV IS 94222, 20. ICMV IS 99001, 21. ICTP 8203, 22. Jirani, 23. KAANATI, 24. LCIC 9702, 25. Moro, 26. Sadore Local, 27. SARIA-1, 28. SOSAT-C88, 29. Souna 3, 30. WAAPP-NAARA.

In the AMMI biplot, the IPCA 1 (PC1) and IPCA 2 (PC2) scores of genotype and environments were plotted against each other for grain yield (Figure 1) and grain Fe content (Figure 2). The IPCA 1 component accounted for 40.26 and 27.54% of G × E interaction, while IPCA 2 accounted for 12.75 and 22.40%, respectively, for grain yield and grain Fe content. In the biplot for grain yield, the genotypes ICMP 177002, IBMV 8402, Souna 3, ICMV IS 85327, and Moro are scattered away from the origin and away from the environmental spikes. Most of the genotypes are close to the origin in the biplot for grain Fe content. The genotypes AFRIBEH-NAARA, ICMV 177001, ICMV 177002, and SOSAT-C88 are scattered away from the origin and also away from the environment spikes.

The AMMI stability value (ASV) ranked the genotypes based on the least score (Table 4). Low scores represent the most stable genotypes across locations. Based on the ASV, the most stable genotype is SOSAT-C88 (ASV = 0.04) followed by LCIC 9702 (ASV = 0.13) for grain yield, ICMV 221 (ASV = 0.05) followed by ICMV IS 99001 (ASV = 0.23) for grain Fe content, and WAAP-NAARA (ASV = 0.28) followed by GB 8735 (ASV = 0.50) for grain Zn content. The sum of mean grain yield and stability rankings (YSI) revealed that the genotypes SOSAT-C88, ICMV IS 99001, and CHAKTI were high yielding and expressed stability across regions. The genotypes ICMV 167001 and ICMV 167005 were high yielding but were unstable, which has a high YSI rank.

**TABLE 4:** Interaction principal component axis scores of agronomic and morphological traits of pearl millet genotypes across locations in West Africa.

**Genotype**	**Grain yield**								**Grain Fe content**				**Grain Zn content**			
	**Mean**	**Rank (X)**	**IPCA 1**	**IPCA 2**	**ASV**	**ASV rank (Y)**	**YSI (X + Y)**	**YSI rank**	**IPCA 1**	**IPCA 2**	**ASV**	**ASV rank**	**IPCA 1**	**IPCA 2**	**ASV**	**ASV rank**
AFRIBEH-NAARA	0.79	19	0.05	−0.11	0.19	3	22	6	1.68	0.88	2.14	28	1.10	−0.01	1.66	18
AKAD-KOM	0.83	17	0.09	0.05	0.26	7	24	8	−1.35	0.27	1.59	26	−0.59	0.00	0.89	7
CHAKTI	1.42	1	0.18	0.16	0.54	15	16	4	0.82	0.21	0.98	16	1.39	−0.36	2.14	25
Faringuero	0.73	21	−0.26	0.08	0.76	18	39	18	−0.15	0.49	0.52	6	−1.43	0.20	2.18	26
Gamoji	1.19	7	−0.05	−0.20	0.25	5	12	2	−0.29	−0.04	0.34	4	−1.02	−0.67	1.68	19
GB 8735	0.83	17	0.05	−0.20	0.25	6	23	7	−0.46	0.48	0.72	10	0.32	0.16	0.50	2
IBMV 8402	0.99	10	−0.59	−0.02	1.71	29	39	18	0.10	0.58	0.59	8	−0.47	−0.92	1.16	14
ICMP 177001	0.85	15	0.28	−0.30	0.86	22	37	16	0.22	−1.38	1.41	22	2.06	0.12	3.11	29
ICMP 177002	1.19	7	0.58	0.25	1.72	30	37	16	−0.20	−0.20	0.31	3	0.69	−1.69	1.99	23
ICMV 167005	1.31	5	−0.19	0.24	0.61	17	22	6	0.32	−0.01	0.37	5	−0.88	0.79	1.55	17
ICMV 167006	1.13	8	−0.29	0.34	0.92	23	31	13	0.89	0.37	1.10	18	0.60	1.86	2.06	24
ICMV 177001	0.61	23	0.14	0.12	0.44	12	35	15	−3.24	1.46	4.04	30	−2.08	1.23	3.38	30
ICMV 177002	0.83	17	0.09	−0.10	0.28	8	25	9	−1.56	−2.19	2.85	29	−0.48	−0.99	1.23	15
ICMV 177003	0.83	17	0.17	−0.21	0.54	16	33	14	0.61	−0.29	0.77	12	0.47	1.58	1.73	20
ICMV 177004	0.80	18	−0.10	0.11	0.32	10	28	11	1.19	−0.26	1.41	23	0.37	0.46	0.73	4
ICMV 221	0.79	19	0.31	−0.14	0.92	24	43	20	−0.01	−0.05	0.05	1	0.33	0.78	0.93	9
ICMV 167001	1.33	3	0.27	0.25	0.83	20	23	7	0.16	0.70	0.72	11	−1.92	−0.74	2.99	28
ICMV IS 85327	0.97	12	−0.29	−0.41	0.94	26	38	17	1.01	−0.10	1.17	19	0.41	−0.08	0.63	3
ICMV IS 94222	1.28	6	−0.17	0.17	0.52	14	20	5	0.41	0.21	0.52	7	−0.24	1.10	1.16	13
ICMV IS 99001	1.32	4	−0.10	−0.01	0.30	9	13	3	0.15	0.15	0.23	2	−0.59	−0.37	0.97	11
ICTP 8203	0.75	20	0.11	0.02	0.33	11	31	13	0.40	−0.69	0.83	13	1.49	0.68	2.35	27
Jirani	0.84	16	0.08	−0.09	0.25	4	20	5	−0.25	−1.20	1.24	21	0.58	0.33	0.93	10
KAANATI	0.69	22	0.37	−0.19	1.08	27	49	22	−0.50	1.35	1.47	24	0.13	−0.79	0.82	5
LCIC 9702	0.87	14	−0.03	−0.10	0.13	2	16	4	0.20	−0.86	0.89	14	1.19	−0.48	1.86	22
Moro	1.06	9	−0.25	−0.40	0.84	21	30	12	0.19	−0.97	1.00	17	0.38	−1.65	1.75	21
Sadore Local	0.73	21	−0.32	0.15	0.93	25	46	21	−0.47	0.46	0.71	9	−0.79	−0.39	1.25	16
SARIA-1	0.61	23	0.27	0.16	0.79	19	42	19	−0.98	−1.79	2.13	27	0.22	−0.86	0.92	8
SOSAT-C88	1.38	2	0.01	0.01	0.04	1	3	1	0.46	0.75	0.92	15	−0.76	0.10	1.15	12
Souna 3	0.98	11	−0.55	0.13	1.62	28	39	18	1.00	0.19	1.19	20	−0.31	0.76	0.89	6
WAAPP-NAARA	0.88	13	0.15	0.26	0.50	13	26	10	−0.36	1.49	1.55	25	−0.15	−0.16	0.28	1

*IPCA 1, interaction principal component axis one score; IPCA 2, interaction principal component axis two score; ASV, AMMI Stability Value; YSI, yield stability index.*

Additive main effects and multiplicative interaction analysis identified four high-yielding genotypes in each of the environments (Table 5). CHAKTI performed well and yielded high at Bambey (Senegal), Nioro (Senegal), Kano (Nigeria), Konni (Niger), Bawku (Ghana), and Gampella (Burkina Faso). The genotype ICMV 167005 performed well at Magaria, Sadore in Niger, and Cinzana location of Mali.

**TABLE 5:** First four AMMI selections based on best grain yielding genotypes in each environment.

**Locations**	**Grain yield (t/ha)**	**Effect**	**Rank**			
			**1**	**2**	**3**	**4**
Maradi (Niger)	0.94	−0.6154	ICMV IS 99001	SOSAT-C88	Moro	IBMV 8402
Magaria (Niger)	0.98	−0.5197	ICMV 167005	ICMV IS 94222	SOSAT-C88	ICMV IS 99001
Bambey (Senegal)	0.96	0.0596	CHAKTI	SOSAT-C88	ICMV IS 99001	ICMV 167001
Nioro (Senegal)	1.07	0.367	CHAKTI	ICMV 167001	ICMP 177002	SOSAT-C88
Sadore (Niger)	0.90	−0.2665	ICMV 167005	CHAKTI	ICMV IS 94222	ICMV 167001
Kano (Nigeria)	0.70	0.1276	CHAKTI	ICMV 167001	ICMV 167005	SOSAT-C88
Konni (Niger)	0.85	0.4307	CHAKTI	ICMV 167001	ICMP 177002	SOSAT-C88
Bawku (Ghana)	0.94	0.5633	CHAKTI	SOSAT-C88	ICMP 177002	ICMV 167001
Gampela (Burkina Faso)	1.06	0.5125	CHAKTI	ICMP 177002	ICMV 167001	SOSAT-C88
Cinzana (Mali)	1.20	−0.6591	ICMV 167005	ICMV IS 99001	ICMV 167001	SOSAT-C88
Best candidate variety			CHAKTI	SOSAT-C88 & ICMV167001	ICMV 167001 & ICMP177002	SOSAT-C88

The phenotypic correlation patterns of micronutrient traits of pearl millet indicated the presence of a highly significant and positive correlation between grain Fe and Zn contents (*r* = 0.98; *P* < 0.01) (Table 6). The grain Fe and Zn contents exhibited significant and negative correlations with days to 50% flowering, plant height, and panicle length and a significant positive correlation with panicle circumference. It is observed that both these micronutrients are not correlated with grain yield (Table 6).

**TABLE 6:** Association of agronomic and morphological traits of pearl millet (*Pennisetum glaucum* L.) across locations in West Africa.

**Traits**	**Downy mildew damage%**	**Days to 50% flowering**	**Plant height**	**Panicle length**	**Panicle circumference**	**Grain yield**	**Grain Fe content**	**Grain Zn content**
Downy mildew damage%	1.00							
Days to 50% flowering	−0.38*	1.00						
Plant height	−0.44*	0.93**	1.00					
Panicle length	–0.20	0.68**	0.68**	1.00				
Panicle circumference	0.31	−0.62**	−0.68**	−0.58**	1.00			
Grain yield	–0.13	0.02	0.08	0.33	0.10	1.00		
Grain Fe content	0.45*	−0.77**	−0.74**	−0.70**	0.55**	0.01	1.00	
Grain Zn content	0.39*	−0.77**	−0.75**	−0.70**	0.57**	0.01	0.98**	1.00

*,**Significant at P = 0.05 and 0.01, respectively.

## Discussion

Micronutrient malnutrition is a major concern in West and Central Africa. Breeding OPVs with grain Fe and Zn micronutrients will be helpful in combating the dietary-based malnutrition among the poor households in this region. Significant differences for grain Fe and Zn densities and grain yield indicate the presence of variability for these traits in the material tested. Environment is one of the major factors that influences the performance of the genotypes. As G × E interaction is important for any trait breeding, ANOVA revealed a higher magnitude of mean squares for genotypes than the mean squares of G × E interaction, marking the lower impact of the environment on the inheritance of these agronomic and micronutrient traits studied across locations.

In the Sahelian zone of West Africa, the farmers prefer to have the early maturing genotypes with long panicles and dual-purpose pearl millet where the rainfall is 300–400 mm (Blümmel et al., 2003; Omanya et al., 2007; Anna Ida Pucher, 2018). In this study, most of the genotypes exhibited early flowering, which is very important for drought-prone areas of Sub-Saharan Africa, especially in the Sahelian zone of West Africa where millet is grown in around 8 million ha out of 16 million ha of total millet cultivation in Africa (Zakari et al., 2019).

CHAKTI and ICMP 177002 have performed well with high grain yield and grain Fe and Zn contents. CHAKTI has iniadi background, which was developed using the intrapopulation method after four cycles of recurrent selections for high Fe. CHAKTI is proposed for the large-scale farmer trials and release in WCA. Previous studies reported that iniadi germplasm has been found to be the best source for grain Fe and Zn contents (Velu et al., 2011; Rai et al., 2014; Govindaraj et al., 2020). The identification of new sources of grain Fe and Zn is equally important as depending on one type of germplasm may be counterproductive in achieving micronutrient genetic gains in pearl millet.

The AMMI ANOVA also indicated the broad range of diversity that existed among genotypes, and the significance of environments and their interactions indicated that there was a differential response of genotypes to environments and will help breeder in choosing genotypes to a specific environment. The significance of IPCA1 and IPCA2 indicated the presence of a complex, multidimensional variation in the G × E data. The AMMI biplot analysis is a predominant method to find the G × E interaction for different studied traits. Low G × E interaction indicates the stability of the genotypes over the range of environments. A genotype showing high positive interaction in an environment can exploit the agroecological or agromanagement conditions of the specific environment and is, therefore, best suited to that environment. Heritability across the locations is very significant indicating a relatively lesser G × E influence; thus, simple selection for micronutrients is highly feasible. These magnitudes of heritability are affected by the type of genetic material, traits variability, and environmental conditions to which the trials are conducted (Dabholkar, 1992; Falconer and Mackay, 1996). Therefore, the heritability estimate is relatively high for grain yield due to conducive rainfall and other environmental conditions such as the adaptability of the new variety.

The most powerful interpretive tool in the analysis of G × E interaction in the AMMI model is the biplot analysis. The biplots permit easy visualization of differences in interaction effects. In the AMMI biplot, the genotypes with high mean performance and large value of IPCA scores are conceived as specific adaptability to the environment. Purchase et al. (2000) proposed an ASV measure to quantify and classify genotypes according to their *per se* potential. The ASV has identified the genotype CHAKTI as the most prominent genotype that outperformed across the majority of the locations, indicating this as a stable performing genotype across environments. The ASV parameter has been successfully used in several studies to find stable performers (Mallikarjuna et al., 2015; Govindaraj et al., 2018). CHAKTI is the top-ranking OPV that had a lower ASV of 0.5–2.0 for grain yield and micronutrients, and still, it ranked first in 6 out of 10 locations and second in only 1 location. Based on the multilocational testing and superior performance, recently in 2018, the first biofortified pearl millet variety “CHAKTI” in Africa was released in Niger (Jayashree and Meyer, 2018) and the ECOWAS regional catalog in 2019^1^. This variety is gaining importance in the farming community in Niger, Mali, Burkina Faso, and Senegal due to its extra early maturity, high yield, and high grain Fe and Zn contents (Mavindidze, 2020).

Association studies among traits revealed the presence of a significant positive correlation between the grain Fe and Zn contents, indicating that they are inherited together and both traits can be selected in every generation. Similar results were earlier reported in pearl millet (Govindaraj et al., 2013; Anand et al., 2014; Govindaraj et al., 2020). This high correlation may be due to the overlapping and co-segregating quantitative trait loci reported in pearl millet (Kumar, 2011), implying that selecting high-Fe lines would likely increase Zn as an associated trait. Grain Fe and Zn contents have exhibited no significant correlation with grain yield, which implies that there is no yield limitation in increasing micronutrients in pearl millet. Earlier studies in pearl millet reported a significant negative correlation with grain yield (Rai et al., 2012; Anand et al., 2014) and no correlations (Govindaraj et al., 2019; Pujar et al., 2020), indicating that conscious selection for both traits is required in segregating population to avoid any negative linkages. Also, it is important to develop the experimental population involving diverse high-yield and high-Fe/Zn parents to study the linkage between yield and nutrient traits in the segregating population or by using genomic tools.

## Conclusion

The biofortification in pearl millet intends to contribute towards the prevention of micronutrient deficiencies by increasing the daily adequacy of micronutrient intakes among the poor farming population in the semi-arid regions. A stable performing high-Fe variety “CHAKTI” was identified across 10 locations, with high grain yield (71% higher) and grain Fe (16% higher) and Zn (7.4% higher) contents than the currently growing OPVs (35 ppm mean Fe and 28 ppm mean Zn). The correlation between Fe and Zn and no correlation with yield indicate that both micronutrients can be improved simultaneously in high-yielding backgrounds. Further study is needed to explore the progeny test-based selections from the identified high-Fe/Zn OPVs for breeding hybrid parents prospecting biofortification in WCA with competitive yield in near future.

## Data Availability Statement

The original contributions presented in the study are included in the article/supplementary material, further inquiries can be directed to the corresponding authors.

## Author Contributions

PG and MG conceived the concept and provided the genetic materials. MR and PG helped in designing the trial, data curation, conducted the research, statistical data analyses, and drafting the manuscript. DI conducted trials in Burkina Faso and contributed to the data analysis and data curation. OS conducted trials in Senegal and contributed to the data curation and data analysis from Senegal. KAI helped in conducting multisite data from Niger. PAA contributed to conducting the trial and data curation from the Ghana trial. MDS helped in conducting the trial and data curation from Mali locations. AII conducted trials in Nigeria and contributed to the data curation from Nigeria. All authors read and agreed to the content of the manuscript.

## Conflict of Interest

The authors declare that the research was conducted in the absence of any commercial or financial relationships that could be construed as a potential conflict of interest.

## Publisher’s Note

All claims expressed in this article are solely those of the authors and do not necessarily represent those of their affiliated organizations, or those of the publisher, the editors and the reviewers. Any product that may be evaluated in this article, or claim that may be made by its manufacturer, is not guaranteed or endorsed by the publisher.

## Footnotes

1
http://www.coraf.org/paired/wp-content/uploads/2019/11/Catalogue-Re%CC%81gional-des-Espe%CC%80ces.pdf
